# A prototype device of microliter volume voltammetric pH sensor based on carbazole–quinone redox-probe tethered MWCNT modified three-in-one screen-printed electrode

**DOI:** 10.1038/s41598-021-93368-5

**Published:** 2021-07-06

**Authors:** Sakthivel Srinivas, Krishnan Ashokkumar, Kamaraj Sriraghavan, Annamalai Senthil Kumar

**Affiliations:** 1 Nano and Bioelectrochemistry Research Laboratory, Carbon Dioxide Research and Green Technology Centre, Vellore Institute of Technology, Vellore, 632 014 India; 2 Department of Chemistry, School of Advanced Sciences, Vellore Institute of Technology, Vellore, 632 014 India

**Keywords:** Electrochemistry, Diagnosis

## Abstract

As an alternate for the conventional glass-based pH sensor which is associated with problems like fragile nature, alkaline error, and potential drift, the development of a new redox-sensitive pH probe-modified electrode that could show potential, current-drift and surface-fouling free voltammetric pH sensing is a demanding research interest, recently. Herein, we report a substituted carbazole-quinone (Car-HQ) based new redox-active pH-sensitive probe that contains benzyl and bromo-substituents, immobilized multiwalled carbon nanotube modified glassy carbon (GCE/MWCNT@Car-HQ) and screen-printed three-in-one (SPE/MWCNT@Car-HQ) electrodes for selective, surface-fouling free pH sensor application. This new system showed a well-defined surface-confined redox peak at an apparent standard electrode potential, *E*^o^′ = − 0.160 V versus Ag/AgCl with surface-excess value, Γ = 47 n mol cm^−2^ in pH 7 phosphate buffer solution. When tested with various electroactive chemicals and biochemicals such as cysteine, hydrazine, NADH, uric acid, and ascorbic acid, MWCNT@Car-HQ showed an unaltered redox-peak potential and current values without mediated oxidation/reduction behavior unlike the conventional hydroquinone, anthraquinone and other redox mediators based voltammetry sensors with serious electrocatalytic effects and in turn potential and current drifts. A strong π–π interaction, nitrogen-atom assisted surface orientation and C–C bond formation on the graphitic structure of MWCNT are the plausible reasons for stable and selective voltammetric pH sensing application of MWCNT@Car-HQ system. Using a programed/in-built three-in-one screen printed compatible potentiostat system, voltammetric pH sensing of 3 μL sample of urine, saliva, and orange juice samples with pH values comparable to that of milliliter volume-based pH-glass electrode measurements has been demonstrated.

## Introduction

**Figure 1:**
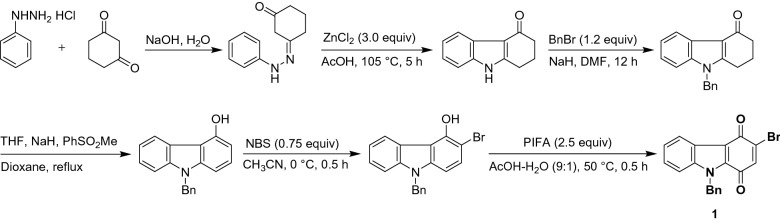
Synthesis route adopted to prepare 9-benzyl-3-bromo-1*H*-carbazole-1,4(9*H*)-dione (Car-HQ) sample.

**Figure 2:**
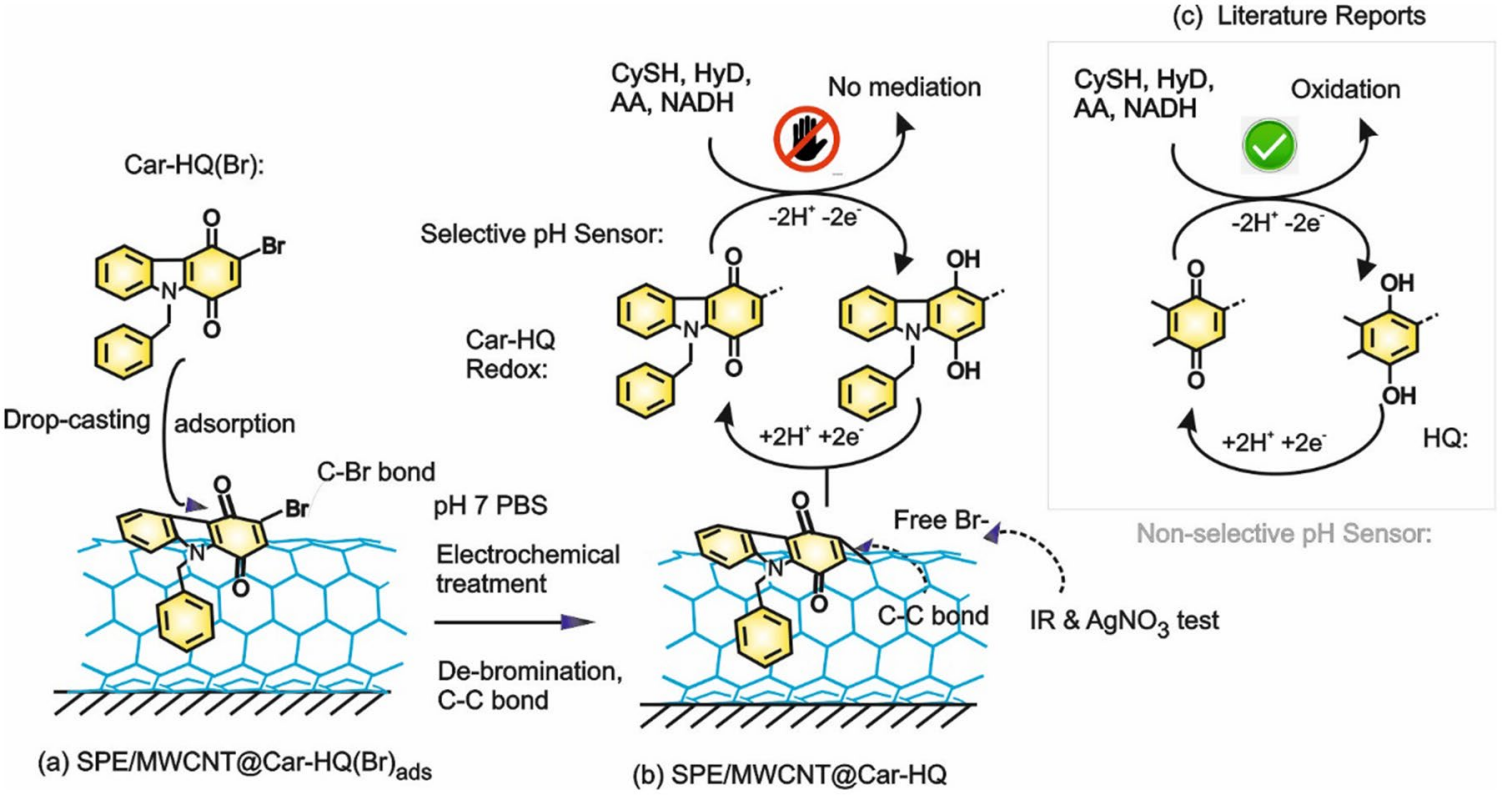
Illustration of preparation of Car-HQ immobilized MWCNT modified electrode by electrochemical approach (**a**, **b**) and its proton-coupled electron-transfer feature without any electrochemical/chemical/electrocatalytic interference from various biochemicals unlike the conventional literature-based reports with hydroquinone and derivatives (**c**).

The determination of solution pH is one of the fundamental analyses that is related to several practical applications including biomedical, clinical, industrial, environmental pollution, food processing, agricultural, pharmaceutical systems etc^1–3^. For instance, salivary pH determination is a diagnostic biomarker for the periodontal disease and oral health status^4, 5^, fruit juice pH sensing is an indicator for the disorientation of pasteurized shelf-stable fruit juices due to the dissolved oxygen coupled degradation of ascorbic acid (AA) and sugar degradation in aqueous solutions^6^, and urinary pH analysis is a biomarker for diabetes mellitus^7, 8^, and urinary tract infections^9^, etc. Although there are several spectroscopic-based pH determination methods like colorimetry^10, 11^, fluorescence^12, 13^, nuclear magnetic resonance^14^, reported, in consideration with portability, on-filed analysis, off-line sample-treatment procedure and user-friendly approach, those methods are not suitable for extension to real-practical applications. In this connection, a wide range of electrochemical pH sensors based on potentiometric, voltammetric and amperometric techniques have been reported^1, 3, 15–17^. Amongst them, a potentiometric-pH sensor based on a bulb-tip glass electrode of diameter 1–12 mm has been frequently used owing to its sensitivity and selectivity, commercial availability, and fast response^1, 3^. Indeed, problems associated with fragile nature, alkaline errors, instability and potential drift are restricting it for specific applications^1, 3^. Similarly, for the amperometric pH sensor case, current-drift due to the facile electrochemical and electrocatalytic oxidation/reduction reactions of co-existing electroactive chemicals/biochemicals like AA, uric acid (UA), cysteine (CySH), NADH, hydrazine (Hyd) and dissolved oxygen etc.^13^, is a serious issue. Alternately, pH-sensitive redox probes, like hydroquinone (HQ)^18, 19^, anthraquinone (AQ)^20, 21^, phenanthroline–quinone^22, 23^, quinone functionalized graphitic edge-planes^24–27^ etc., based voltammetric pH-sensing approach have been reported as an alternate choice. Nevertheless, the part of the above-mentioned problems like potential drift due to the mediated electrochemical oxidation and reduction reactions by the redox probes (Table 1) couldn’t be completely eliminated^1, 3, 25^. Thus, the development of a specific redox probe suitable for interference-free selective voltammetric pH sensor application is a challenging task. Herein, we introduce, a synthetically prepared benzyl and bromo substituted carbazole-quinone (Fig. 1) immobilized MWCNT modified screen-printed electrode, designated as SPE/MWCNT@Car-HQ as an efficient and interference-free redox-probe system for elegant voltammetric pH sensing applications (Fig. 2).

**Table 1:** Comparison of electroanalytical data of pH sensing by SPE/MWCNT@Car-HQ with several literature-based voltammetric sensing systems.

S. no.	Redox mediator	CME	*E*^o^/mV versus Ag/AgCl	Slope/mV pH^−1^	Interf	Refs.
1.	Dopamine	Au@Dopamine-SAM	–	59 ± 0.05	NADH	^38^
2.	α-Naphthol	GCE/MWCNT@np-quinone	− 100 ± 5	80	NADH, Hyd	^39^
3.	Quinoline	GCE/MWCNT@QLO	− 450 ± 5	64	Hyd	^40^
4.	Pyrroloquinoline quinone	GCE/fWCNT	+210	–	Hyd	^41^
5.	Hematin	GCE/GMC/Hemt@Chit	− 390	− 32	DO, H_2_O_2_	^42^
6.	Hemoglobin	GCE/GMC/Heme-Nf	− 380	30 ± 0.12	H_2_O_2_	^43^
7.	Carbazole	GCE/MWCNT@Car	− 215 ± 5	− 59	Hyd	^44^
8.	Anthraquinone	GCE/AQ@f-MWCNT	− 285	− 58	DO, H_2_O_2_	^45^
9.	This work	SPE/MWCNT@Car-HQ	− 160	− 48.1	No interference	

*DO* dissolved oxygen, *Hyd* hydrazine, *Car* carbazole, *Ppy* polypyrrole, *GMC* graphitized mesoporous carbon, *SAM* self assembeled monolayer.

## Results and discussion

### Design and development of Car-HQ modified carbon nanomaterial

**Figure 3:**
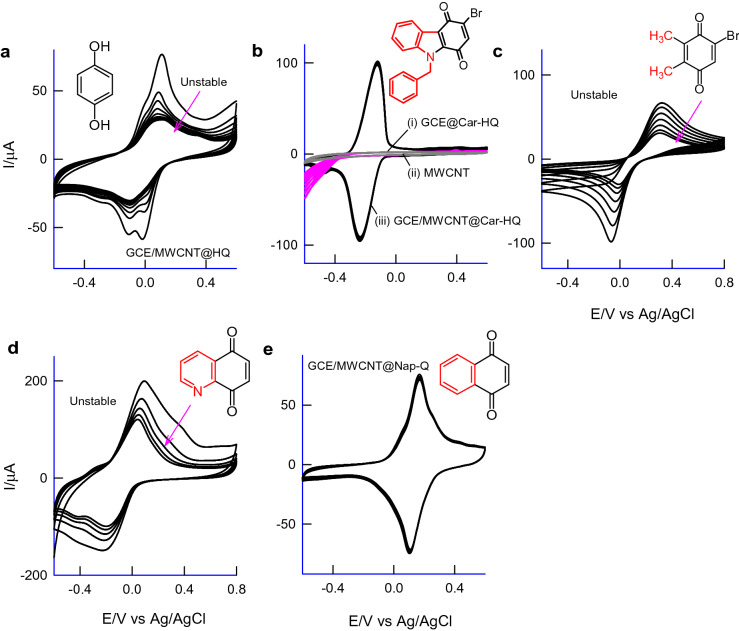
Ten continuous CV responses of various quinone-based redox-active organic compounds immobilized MWCNT modified glassy carbon electrodes (**a**–**e**) in pH 7 phosphate buffer solution at *v* = 50 mV s^−1^. Insets figures are respective redox-active organic compounds used for immobilization.

Since, there are some stability (Fig. 3a) and selectivity complications due to the redox-mediated electrocatalytic oxidation of certain biochemicals like NADH, Hyd and CySH, HQ-based chemically modified electrodes has been rarely used for reagent-less pH-sensing applications^1, 3^. In fact, covalently functionalized AQ-chemically modified carbon electrodes were also showed the stability problem^23^. Thus, in this report following effort was taken to design a new redox-probe molecule that can be immobilized on graphitic carbon surface and can be used effectively; (i) HQ with increased aromatic units: An additional aromatic ring may provide a strong π–π interaction between the aromatic πe^−s^ and *sp*^2^ carbon of graphitic structure for improvement of the stability. (ii) Introduction of a labile halogen atom (C–Br) on the molecular structure: Aim of this part of the work is to generate potential assisted carbo-cation, > C(+) and the nucleophilic addition of MWCNT resulted in the Carbon–Carbon linked new redox-mediated modified electrode surface. (iii) Introduction of a nitrogen atom: It may provide different adsorption and spatial orientation of the redox-active organic molecule on the graphitic surface^28, 29^, and in turn to interference-free voltammetric pH sensor application. By keeping all these points, a new redox-active organic compound, Car-HQ has been designed rationally and synthesized (Fig. 1 and Supporting info) for our electroanalytical studies (Fig. 2).

**Figure 4:**
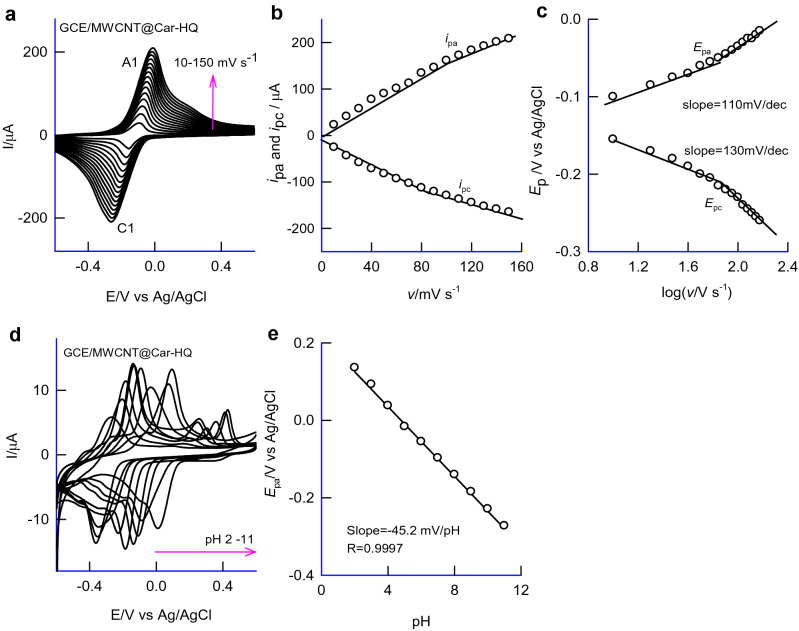
(**a**) CV response of GCE/MWCNT@Car-HQ at various scan rates, *v* = 10–150 mV s^−1^ in pH 7 PBS, (**b**) plot of (*i*_pa_ and *i*_pc_) versus scan rate and (**c**) plot of (*E*_pa_/V) versus log (*v*/V s^−1^). (**d**) is the CV response of GCE/MWCNT@Car-HQ at different pHs and (**e**) its corresponding calibration plot.

Figure 3b curve-(i) is a ten continuous CV responses of Car-HQ adsorbed GCE in pH 7 PBS, showing a feature-less volumetric response. Interestingly, when the same experiment was performed on MWCNT modified GCE, GCE/MWCNT, a well-defined redox peak at an apparent standard electrode potential, *E*^o^′ = − 0.160 ± 0.005 V versus Ag/AgCl with a peak-to-peak separation value, Δ*E*_p_ = *E*_pa_–*E*_pc_, wherein, *E*_pa_ and *E*_pc_ are anodic and cathodic peak potentials, 50 mV (at *v* = 10 mV s^−1^; 150 mV at *v* = 50 mV s^−1^) and surface-excess, Γ = 47 n mol cm^−2^ were noticed (Fig. 3b and curve-(iii)). The calculated relative standard deviation (RSD) between the 1st and 10th cycles of the CV is 2.1% highlighting a stale voltammetric response of the organic molecular modified electrode. The effect of scan rate on the modified electrode showed a systematic increase in the anodic (*i*_pa_) and cathodic (*i*_pc_) peak current signals with an increase in the scan rate (Fig. 4a). Note that a shoulder-like observation is noticed at about 0.2 V versus Ag/AgCl which may be due to the electron-transfer feature of nitrogen functional group Car-HQ. A plot of *i*_pa_ and *i*_pc_ versus scan rate is linear ascribing adsorption-controlled reaction mechanism for the electron-transfer reaction (Fig. 4b).

The kinetics parameters such as transfer coefficient, α and heterogenous electron-transfer rate constane, *k*_s_ were calculated using following Laviron model equations for the surface-confined redox process system with Δ*E*_p_ > 200 mV (Fig. 4c)^30, 31^.

1 $${\text{Sl}}_{{\text{a}}} /{\text{Sl}}_{{\text{c}}} = {\text{ }}\alpha /{\text{1}} - \alpha$$

2 $$\begin{aligned} {\text{log}}k_{{\text{s}}} & = {\text{ }}\alpha {\text{ log }}\left( {{\text{1}} - \alpha } \right){\text{ }} + {\text{ }}\left( {{\text{1}} - \alpha } \right){\text{ log }}\alpha {\text{ }} - {\text{ log }}\left[ {RT/nFv} \right]{\text{ }} \\ & \quad {-}{\text{ }}\alpha {\text{ }}\left( {{\text{1}} - \alpha } \right)nF\Delta E_{{\text{p}}} /{\text{ 2}}.{\text{3}}RT \\ \end{aligned}$$

wherein Sl_a_ is the slope of the linear plot of *E*_pa_ versus log *v* (0.110 V), Sl_c_ is the slope of the linear plot of *E*_pc_ versus log *v* (0.130 V), *v* is applied scan rate (0.05 V s^−1^) and Δ*E*_p_ is a peak separation (0.300 V). The α and *k*_s_ were calculated to be 0.46 and 0.48 s^−1^ respectively. This *k*_s_ value (0.48 s^−1^) is comparable with values obtained for organic-redox probes chemically modified electrodes such as MWCNT@Ellagic acid (3.42 s^−1^)^31^ and GCE/MWCNT@HQ (1.31 s^−1^)^32^. Similarly, the α (0.46) is close to an ideal value (0.5) for a symmetrical energy barrier electron-transfer reaction^33^.

Figure 4d is an effect of solution pH (2–11) on the CV response of the GCE/MWCNT@Car-HQ. A regular variation in the redox potentials, *E*_pa_ and *E*_pc_ against changes up to pH 11 after that plateau in the response was noticed indicating a limitation in the proton-coupled electron-transfer mechanism of the redox reaction in the window, pH 2–11. A pKa (~ pH = 13) of the Car-HQ is the likely reason for the termination in the *E*_pa_ value^27^. A plot of *E*_pa_ versus pH showed a linear line in a window, pH 2–11 with a slope and regression coefficient values of − 45.2 ± 1 mV pH^−1^ and 0.9997 respectively (Fig. 4e). The obtained slope value reminiscent involvement of the non-Nernstian H^+^/e^−^ transfer feature (2H^+^/3e^−^) of the redox mechanism. The tethered nature of the Car-HQ, its surface orientation, and partial protonation of the N-atom of Car-HQ are likely the influencing parameters for the observation^34^. Similar kind of Non-Nernstian observation was previously reported with various proton-coupled electron transfer systems such as Hemoglobin(Hb)/Nafion/MWCNT modified electrode (− 50.9 mV pH^−1^)^35^ and redox-active quinolone-quinones on MWCNT modified electrode (− 54 mV pH^−1^)^36^. Indeed, the *E*_pa_ versus pH linearity is found to be highly suitable to extend for practical applications.

### Physicochemical characterization

**Figure 5:**
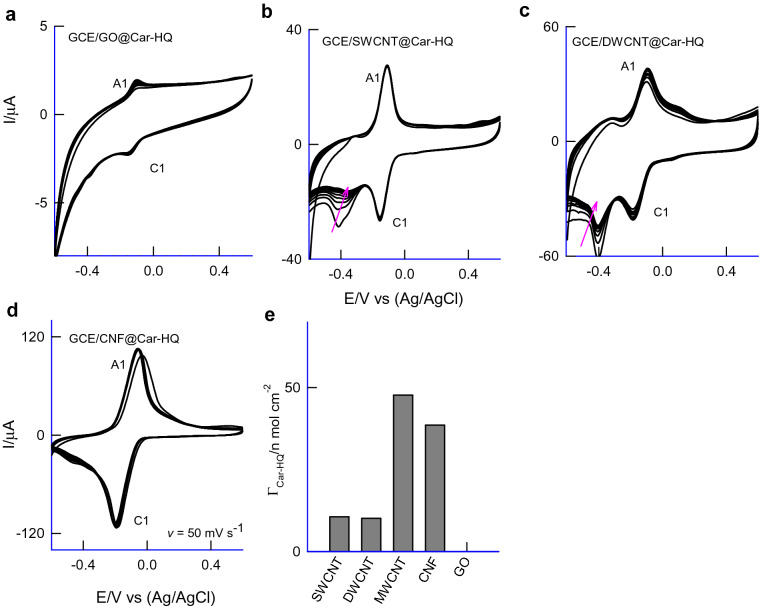
Comparative CV responses of Car-HQ modified various carbon materials; (**a**) GCE/GO@Car-HQ, (**b**) GCE/SWCNT@Car-HQ, (**c**) GCE/DWCNT@Car-HQ, (**d**) GCE/CNF@Car-HQ in pH 7 phosphate buffer solution at *v* = 50 mV s^−1^. (**e**) A bar diagram of surface excess value versus various carbon material modified Car-HQ systems.

**Figure 6:**
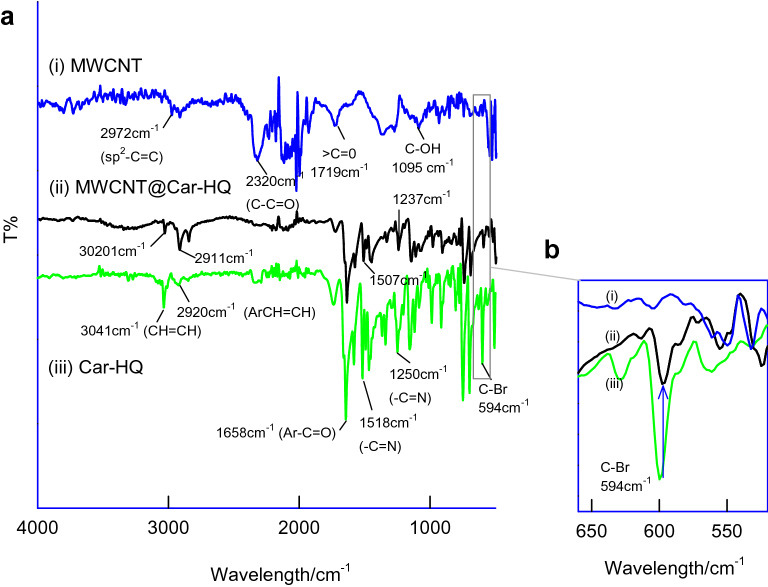
(**a**) FTIR/KBr responses of MWCNT (i), (ii) MWCNT@Car-HQ and (iii) Car-HQ samples and (**b**) Enlarged FTIR responses of the sample at a limited region.

To understand the Car-HQ molecular interaction with carbon surface, various carbon nanomaterials of different features like single-walled carbon (SWCNT), double-walled carbon (DWCNT), graphene-oxide (GO; contains rich oxygen functional group) and carbon nanofiber (CNF), graphitized mesoporous carbon (GMC; structure similar to the carbon nanotube, without any hollow structure arrangement) were subjected to Car-HQ adsorption and electrochemical performance. Figure 5a–e is ten continuous CV responses of SWCNT@Car-HQ, DWCNT@Car-HQ, GO@Car-HQ, MWCNT@Car-HQ and CNF@Car-HQ in pH 7 PBS. Except for GO, all other carbon nanomaterial-modified electrodes showed qualitatively similar voltammetric responses. Based on anodic current, *i*_pa_ value the decreasing order of redox process (RSD) is arranged as CNF (graphitized) (4.5%) > MWCNT (2.1%) > SWCNT (2%) > DWCNT (9%) > GO (25%). Following conclusions can be obtained from the results: (i) Graphitic structure is necessary for the strong π–π assisted immobilization of Car-HQ. (ii) Oxygen functional group in the GO may hinder the immobilization. Presumably, repulsive interaction between the electronic structure of quinone oxygen and graphitic oxygen destabilize the adsorbed Car-HQ on GO. (iii) Considering the relative stability, CNF based material showed poor working stability and MWCNT is found to be more suitable for Car-HQ immobilization and pH sensor application. Figure 6a is a comparative FTIR response of Car-HQ, MWCNT and MWCNT@Car-HQ samples. The Car-HQ showed characteristic IR signals for –CH=CH– (3041 cm^−1^), Ar–C=O (1658 cm^−1^), –C=N (1518, 1250 cm^−1^) and C–Br (594 cm^−1^) similarly MWCNT for *sp*^2^ bonding, –C=C– (2972 cm^−1^), –C–C=O (2320 cm^−1^, fraction of CO_2_ is adsorbed)), > C=O (1719 cm^−1^) and –C–OH (1095 cm^−1^). When the above signals were compared with IR of the MWCNT@Car-HQ, most of the peaks were found to be retained but a significant shift in the frequencies depicting that the Car-HQ is strongly bonded on the graphic sites of MWCNT. In further, the signal of νC-Br in Car-HQ is markedly diminished after the electrochemical preparation of MWCNT@Car-HQ. The C–Br site of Car-HQ might be involved in the C–C bond formation with cleavage of the C–Br bond upon the electrochemical conditioning of the modified electrode. To confirm the free Br^−^ ion, the modified electrode was exposed with a dilute solution of AgNO_3_. It results in the formation of a white precipitate of AgBr which indicates the cleavage of C–Br bond that strongly supports a plausible Carbon–Carbon bond formation between Car-HQ and MWCNT (Supplementary Information Fig. S1). The parallel control experiment without pre-electrochemical treatment of GCE/MWCNT@Car-HQ did not furnish any precipitation which indicates the absence of free Br^−^ ion. Based on the results, it is proposed that the Car-HQ get strongly adsorbed on the graphitic structure of MWCNT via π–π interaction and fraction of C–C bond formation. It is believed that the attachment of N-benzyl-indole moiety of Car-HQ strongly supports the improved π–π tethering on the graphite structure^37^. For control experiments (Fig. 3), Car-HQ derivative compounds without the indole moiety were chosen namely, hydroquinone (Fig. 3a), 5-bromo-2,3-dimethylcyclohexa-2,5-diene-1,4-dione (Fig. 3c), quinoline-5,8-dione (Fig. 3d) and naphthalene-1,4-dione (Fig. 3e) and subjected to immobilization on MWCNT similar to the preparation condition applied for MWCNT@Car-HQ. Figure 3c, d are typical CV responses of the control samples showing unstable voltammetric responses (slow deterioration of the electroactive species) due to weak π-tethering interaction, unlike MWCNT@Car-HQ case. In the case of Nap-HQ (Fig. 3e), a stable voltammetric response was observed along with an efficient mediated oxidation of AA which is the prime cause of the interference (Supplementary information Fig. S2) and in inturn to the potential and current drifts with voltammetry pH sensor systems.

### Voltammetric pH sensor

**Figure 7:**
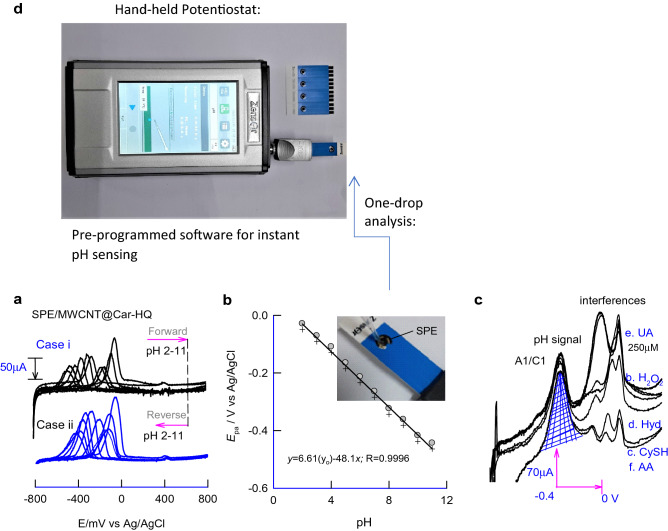
DPV responses of SPE/MWCNT@Car-HQ modified with one drop of; (**a**) different pHs tested by forward and reverse sweeping and (**c**) with various interfering chemicals. (**b**) is a corresponding plot of *E*_p_ versus pH of the solution. (**d**) Photo graph of hand-held potentiostat compatible for SPE and preprogrammed software for instant pH sensing applications. Photographs were taken by the first author.

For practical convenience, DPV based approach has been adopted for the voltammetric pH sensor applications in this work. Figure 7a, cases-1 and II, are typical DPV responses of MWCNT@Car-HQ modified three-in-one screen-printed electrode (SPE), designated SPE/MWCNT@Car-HQ, tested for back and forth pH sensing experiments by systematically increasing the pH of the solution from 2 to 11 as a first set followed by repeating the same in the reverse order, 11–2 as a second set of experiment. Although there is a significant alteration in the peak current responses, the observed peak potential values were nearly the same in the back–forth experiment data. Note that in the above cases, the Car-HQ-layer was modified manually on the carbon-working surface of the SPE. A systematic variation in the redox peak signal against the solution pH was noticed. The obtained redox-peak response was found to be nearly the same in the back and forth voltammetric analyses. Figure 7b is a plot of *E*_pa_ (DPV) versus solution pH showing a linear line fit-with a linear-regression equation and regression coefficient values, *E*_pa_ (mV) = − 48.1 ± 0.9 (mV pH^−1^) pH + 6.61 (*E*^o^/mV) and 0.9996 respectively. The slope value is closer to the response (− 45.2 mV pH^−1^) obtained with pH effect by conventional GCE modified electrode in the CV analysis verifying the applicability of the analytical protocol on the screen-printed electrode surface. In further, the linear-equation data is programmed into the portable potentiostat and tested for instant pH value after performing the voltammetric run with the real sample (Fig. 7d).

Before the real sample analysis, the GCE/MWCNT@Car-HQ was subjected to electrochemical studies with various electroactive chemicals/biochemicals like CySH, Hyd, UA and AA in pH 7 PBS. With all the electroactive chemicals/biochemicals, there is no marked alteration in the potential (*E*_pa_) and the current signal of the redox peak (base-line corrected *i*_pa_) unlike the pH-sensitive redox probes like HQ, AQ and Nap-HQ (Supplementary Information Fig. S2), all of which showed marked electrocatalytic interferences and inturn potential and current drifts (Table 1)^38–45^. Note that the electrochemical oxidation and reduction-interference signals were noticed only at 0.1–0.4 V regions where there is no influence of the A1/C1 redox peak (Fig. 6c). This is a novel observation of this work to extend into potential and current drift-free voltammetric pH sensing applications.

**Figure 8:**
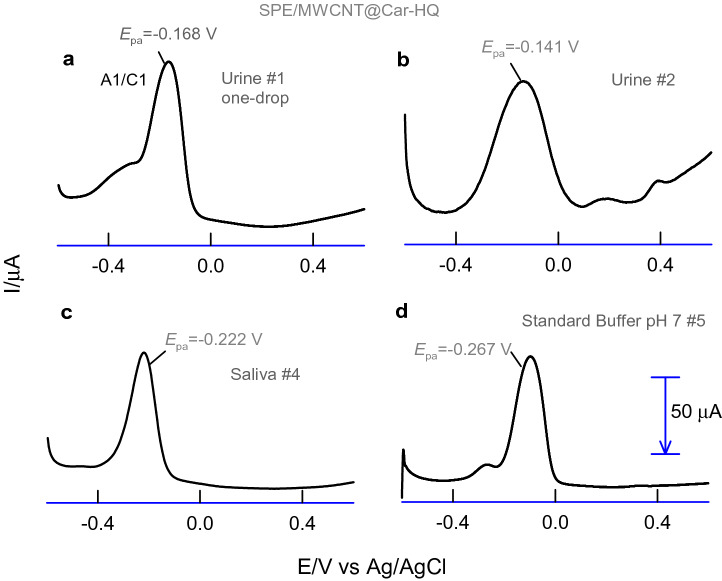
DPV responses of SPE/MWCNT@Car-HQ modified with one-drop (3 µL) of various real samples (**a**–**d**). #1–#2 = different human urine samples, **c** saliva and **d** standard pH 7 buffer.

Three-in-one screen-printed electrode coupled voltammetric analyses of the real sample were presented in Fig. 8a–d. A photograph of the online pH measurement using the portable and programmed potentiostat is shown in Fig. 7b, d. As displayed in the pictures, 3 μL volume of a raw-real solution (saliva) was dropped on the SPE surface using a micropipette and subjected to ‘online pH sensing’. Based on the voltammetry peak potential and in-built software program, the respective solution pH was able to measure within 15 s of the experimental time. Also, a parallel pH measurement of the milliliter volume of the test samples was carried out using a conventional pH meter. Figure 8 showed typical one-drop pH sensing responses of various real samples. Table 2 provides comparative pH values obtained by the micro-liter voltammetric pH sensor and conventional pH meter. The error values are in the range of 1–3% validating the accuracy and suitability of our new voltammetric pH sensor for false-free pH sensor practical applications. In further, to test precision, pH measurement of the standard buffer solution of pH 7 was tested repeatedly for eight times with the same electrode showing the *E*_pa_ alteration of 3.1%. Similarly, three different screen-printed electrodes (obtained from different packs) modified with the MWCNT@Car-HQ were also tested for the above-mentioned experiment showing a 9.8% of error in the *E*_pa_ values. Indeed, the analytical performance can be improved if a uniform screen-printed electrode surface and modification procedure will be adopted.

**Table 2:** Three-in-one screen-printed electrode modified with MWCNT@Car-HQ for one drop pH analysis of real sample and comparison data with a standard pH meter.

S. no.	Real sample	*E*_pa_ mV versus Ag-pseudo ref	This work	pH meter	Relative error (%)
1.	Urine, #1	− 0.168	4.88	4.80	1.56
2.	Urine, #2	− 0.141	4.32	4.25	1.47
3.	Urine, #4	− 0.101	3.47	3.52	1.44
4.	Saliva, #4	− 0.222	6.07	6.01	1.01
5.	Std buffer pH 7, #5	− 0.267	6.95	7.01	1.04
6.	Undiluted orange, #6	− 0.121	3.80	3.73	1.63
7.	Watermelon, #7	− 0.208	5.70	5.65	1.03

## Conclusions

A new redox-active quinone containing indole moiety, Car-HQ has been designed, synthesized and explored for voltammetric pH sensor application. The Car-HQ immobilized MWCNT modified GCE and screen-printed three-in-one electrodes, prepared by simple solution phase-drop casting procedure, showed a well-defined redox peak at *E*^o^′ = − 0.160 V versus Ag/AgCl in pH 7 phosphate buffer solution. The redox system has followed an adsorption controlled electron-transfer mechanism with transfer coefficient and heterogeneous electron-transfer values, 0.48 and 1.1 s^−1^ respectively. The strong π–π interaction due to the presence of the multiple aromatic rings, the spatial arrangement of nitrogen attached benzyl group and the formation of carbon–carbon bond due to the electrophilic carbon of C–Br are important parameters for surface-fouling free redox activity of the GCE/MWCNT@Car-HQ chemically modified electrode. A screen-printed three-in-one modified electrode in couple with MWCNT@Car-HQ as a working electrode showed a systematic variation in the redox peak against solution pH tested by the DPV technique. A plot of *E*_pa_ versus pH is linear with slope and regression coefficient values, − 48.1 mV pH^−1^ (non-Nernstian behavior) and 0.9997 respectively. This new redox system showed an unaltered redox peak current and potential behaviors in presence of several electroactive chemicals/biochemicals, unlike the conventional HQ, AQ and Nap-HQ based redox systems which showed marked potential and current drifts due to electrocatalytic oxidation and reduction. Practical pH sensing applicability was tested by placing a 3 μL of real samples of fruit juices, saliva and urine on the SPE/MWCNT@Car-HQ sensor which has been coupled with a pre-programmed portable potentiostat system. The obtained values were closely matching with the values measured by the conventional milliliter volume pH-glass sensor electrode validating the applicability of this new voltammetric pH sensor system.

## Experimental section

### Chemical and materials

Single-walled (SWCNT; ≥ 95% purity on carbon basis, 0.84 nm average diameter), double-walled (DWCNT; < 10% Metal Oxide (TGA); 50–80% purity on carbon basis, outer diameter: 5 nm; inner diameter: 1.3–2.0 nm; length: 50 µm), multi-walled (MWCNT; ≥ 98% purity on carbon basis, outer diameter: 10 nm ± 1 nm; inner diameter: 4.5 nm ± 0.5 nm; length: 3- ~ 6 µm), carbon nanofibers, graphitized (CNF; > 99.9% purity on carbon basis, D × L 100 nm × 20–200 µm), graphene oxide (GO > 80% carbon basis flake size − 0.5–2.0 µm, thickness 0.6–1.2 nm) were purchased from Sigma-Aldrich, USA. A redox-active organic compound, 9-benzyl-3-bromo-1*H*-carbazole-1,4(9*H*)-dione was designed and synthesized as per Fig. 1. The detailed synthetic procedure was presented in the supplementary information file. All the chemical reagents and chemicals were of analytical grade and used as received without any further purification. Aqueous solutions were prepared using deionized and alkaline KMnO_4_ distilled water. Unless otherwise stated, pH 7 phosphate Buffer Solution (PBS) was used as a supporting electrolyte. Other pH solutions, range 2–11 were prepared by using mixing orthophosphoric acid and sodium hydroxide at different ratios. Since there is no dissolved oxygen interference, voltammetric pH sensor measurements were carried out in normal dissolved oxygen-containing electrolyte solutions.

### Instrumentation

Voltammetric measurements were carried out using FRA2 µAutolab, PotentioStat/Galvanostat, electrochemical workstation (Metrohm-Autolab, Netherlands) and Zensor, Simulator sensor (ECAS 100, 2nd generation of PotentioStat, Taiwan) coupled screen-printed electrode instruments. The Zensor simulator provides an in-built software program to feed the linear E-pH equation and for instant pH results. Initial experiments were carried out using a conventional three electrodes system consisting of a glassy carbon electrode (GCE) of 0.0707 cm^2^ geometrical surface area (Tokai, Japan) and its chemically modified form (CME) as a working electrode, Ag/AgCl with 3 M KCl as a reference electrode and platinum wire as a counter electrode. For the pH sensor applications, a Zensor TE100 three-in-one electrode consisting of carbon that was modified with MWCNT@Car-HQ as a working and carbon-layer as a counter and Ag-layer as a reference electrode, was used. A Bio-analytical system (BAS, USA) polishing kit was used to polish the GCE surface. Fourier transform-infrared (FT-IR) spectral (4000–400 cm^−1^) analysis was carried out by using JASCO 4100 Spectrophotometer by the KBr pellet method.

### Preparation of GCE (or SPE)/MWCNT@Car-HQ modified electrode

In first, the surface of GCE was cleaned by mechanical polishing and electrochemical pre-treatment procedures. For the second case, twenty-five potential cycling of GCE (or SPE) in a window, − 0.2–1.0 V versus Ag/AgCl at a scan rate (*v*) of 50 mV/s in PBS 7 solution was performed. A 3 µL of MWCNT-ethanol suspension prepared by dispersing 2 mg of MWCNT nanopowder in 500 µL of absolute ethanol (99.9%) was drop-casted on the cleaned GCE (or SPE) followed by drying at room temperature (T = 30 °C) for 5 ± 1 min. Then, 5 µL of 5 mM of Car-HQ/EtOH solution was drop-casted on the GCE/MWCNT and air-dried. The same procedure was repeated for the preparation of SPE modified Car-HQ electrode. As a pre-treatment procedure, the modified electrode was potential-cycled for twenty-five continuous C versus in a window, − 0.2–1.0 V versus Ag/AgCl at a scan rate (*v*) of 50 mV s^−1^ in PBS 7 solution was performed (Fig. 2).

### Real sample preparation

Human urine and saliva were collected from a healthy 22–25 year-old adults. For all the real sample analyses, 3 µL of the undiluted raw samples were dropped directly on the three-in-one screen-printed electrode surface (SPE/MWCNT@Car-HQ) and performed the differential pulse voltammetric (DPV) response. Following is an optimal condition for the DPV parameters; Incremental potential = 4 mV; amplitude = 50 mV; pulse width = 0.06 s; sample width = 0.02 s; pulse period = 0.5 s.

## Supplementary Information

Supplementary Informations.
