# Supplementary material for: A prototype device of microliter volume voltammetric pH sensor based on carbazole–quinone redox-probe tethered MWCNT modified three-in-one screen-printed electrode
Source: Sci Rep. 2021 Jul 6;11:13905. doi: 10.1038/s41598-021-93368-5 (PMC8260652; doi:10.1038/s41598-021-93368-5)
Supplement: Supplementary file 1 — Supplementary Informations. [file 41598_2021_93368_MOESM1_ESM.pdf]

Supporting Information

A Prototype Device of Microliter Volume Voltammetric pH Sensor Based on Carbazole-Quinone Redox-Probe Tethered MWCNT Modified Three-in-One Screen-Printed Electrode

Sakthivel Srinivas,[a,b] *Krishnan Ashokkumar,*[b] *Kamaraj Sriraghavan,*[b*] *Annamalai Senthil Kumar* [a,b]*

[a] Nano and Bioelectrochemistry Research Laboratory, Carbon dioxide Research and Green Technology Centre, Vellore Institute of Technology, Vellore-632 014, India

[b]Department of Chemistry, School of Advanced Sciences, Vellore Institute of Technology, Vellore-632 014, India

1. Synthesis and characterization of phenyl substituted Car-HQ[40,41]

1.2 Synthesis of (Z)-3-(2-phenylhydrazono)cyclohexan-1-one

To a solution of cyclohexane-1,3-dione (2.00 g, 17.84 mmol) in H_2O (25 mL) was added a filtered solution of phenyl hydrazine hydrochloride (2.58 g, 17.84 mmol) dissolved in combination of H_2O (25 mL) and aqueous solution of NaOH (5 N, 4 mL, 19.62 mmol) slowly over the period of 0.5 h and stirred at r.t. for another 5 h. Then the mixture was quenched with ice-water (200 mL) fine solid was obtained it was filtered off and dried. It affords 3-(2-phenylhydrazono)cyclohexan-1-one as a pale brown solid (3.43 g, 16.94 mmol, 95%).

1.3. Synthesis of 1,2,3,9-tetrahydro-4H-carbazol-4-one

To a solution of zinc chloride (15.16 g, 111.24 mmol) in AcOH (15 mL) was added cyclohexane-1,3-dione monophenylhydrazone (3.00 g, 14.83 mmol) in small portions over 5 min at 70 °C stirring, after completion of addition the reaction mixture was heated at 105 °C for 5 h. Then the mixture was cooled to room temperature and poured into chopped ice fine solid was obtained it was filtered off and dried. It affords 1,2,3,9-tetrahydro-4H-carbazol-4-one as a pale green solid (2.56 g, 13.79 mmol, 93%).

1.4. Synthesis of 9-benzyl-1,2,3,9-tetrahydro-4H-carbazol-4-one

To a solution of 1,2,3,9-tetrahydro-4H-carbazol-4-one (2.50 g, 13.49 mmol) in dry DMF (30 mL) was added NaH (60% dispersion in mineral oil, 0.81 g, 20.25 mmol) in small portions at 0 °C and the mixture was stirred for another 30 min at the same temperature. And the reaction mixture was allowed to warm to the room temperature then benzyl bromide (1.76 mL, 2.54 g, 14.84 mmol) was added and it was stirred for another 12 h and quenched with ice-water (300 mL) fine solid obtained it was filtered, washed with excess amount of water and dried. To afford 9-Benzyl-1,2,3,9-tetrahydro-4*H*-carbazol-4-one as a brown solid (3.31 g, 12.01 mmol, 89%).

1.5. Synthesis of 9-benzyl-9H-carbazol-4-ol

To a solution of 9-Benzyl-1,2,3,9-tetrahydro-4*H*-carbazol-4-one (3.00 g, 10.89 mmol) in dry THF (50 mL) was added NaH (60% dispersion in mineral oil, 1.09 g, 27.24 mmol) in argon atmosphere at r.t. And the mixture was stirred for 30 min and then freshly prepared methyl benzenesulfinate (2.14 mL, 2.55 g, 16.34 mmol) was added. Then the reaction mixture was refluxed for 4 h and cooled to the room temperature and subsequently quenched with water (30 mL) and aqueous solution sat. NH_4Cl (15 mL) the mixture was extracted with EtOAc (5 X 50 mL) and the combined organic layers were dried over $MgSO_4$ and concentrated. The residue was re-dissolved in 1,4-dioxane (30 mL) and it was refluxed for overnight. Then the mixture was cooled to room temperature, the solution was concentrated and the residue was purified by flash chromatography it provides 9-Benzyl-3-bromo-9*H*-carbazol-4-ol as a colorless solid (2.03 g, 7.43 mmol, 68%).

1.6 Synthesis of 9-benzyl-3-bromo-9H-carbazol-4-ol

To a solution of 9-Benzyl-9*H*-carbazol-4-ol (2.00 g, 7.32 mmol) in dry CH_3CN (40 mL) was added a freshly recrystallized NBS (977 mg, 5.50 mmol) at 0 °C and stirred for 0.5 h at the same temperature. And the reaction mixture was concentrated in vacuo and the residue obtained was purified over flash chromatography using hexane-ethyl acetate (80:20) as an eluant to afford 9-Benzyl-3-bromo-1*H*-carbazole-1,4(9*H*)-dione as a colorless solid (2.40 g, 6.80 mmol, 93%).

1.7 Synthesis of 9-benzyl-3-bromo-1H-carbazole-1,4(9H)-dione (1, Car-HQ)

To a solution of 9-Benzyl-3-bromo-9*H*-carbazol-4-ol (2.40 g, 6.81 mmol) in a 9:1 mixture of AcOH and H_2O (30 mL) was added PIFA (7.33 g, 17.0 mmol) in one portion and stirred at 50 °C for 0.5 h. Then, to this MeOH (30 mL) was added and stirring was continued for an additional 0.5 h at the same temperature after that the reaction mixture was quenched with ice-water and extracted with dichloromethane (3×50 mL). The combined organic layers were washed with water, dried over anhydrous $MgSO_4$ and concentrated in vacuo. The residue was purified by flash chromatography to afford 9-benzyl-3-bromo-1H-carbazole-1,4(9H)-dione as red needles (2.27 g, 6.20 mmol, 91%). Mp: 118-120 °C. ^{1}H NMR (400 MHz, $CDCl_3$) δ 8.30 (d, *J* = 8.0 Hz, 1H), 7.45-7.36 (m, 3H), 7.29-7.23 (m, 3H), 7.15-7.10 (m, 3H)5.82 (s, 2H); ^{13}C NMR (100.6 MHz, $CDCl_3$) δ 178.0, 175.0, 140.2, 139.1, 137.2, 135.9, 133.1, 128.8, 128.0, 127.5, 126.7, 125.1, 124.3, 123.2, 116.2, 111.7, 48.2; IR (neat) 1643, 1516, 1249, 1155, 748, 698 cm^{-1}.

A. B.

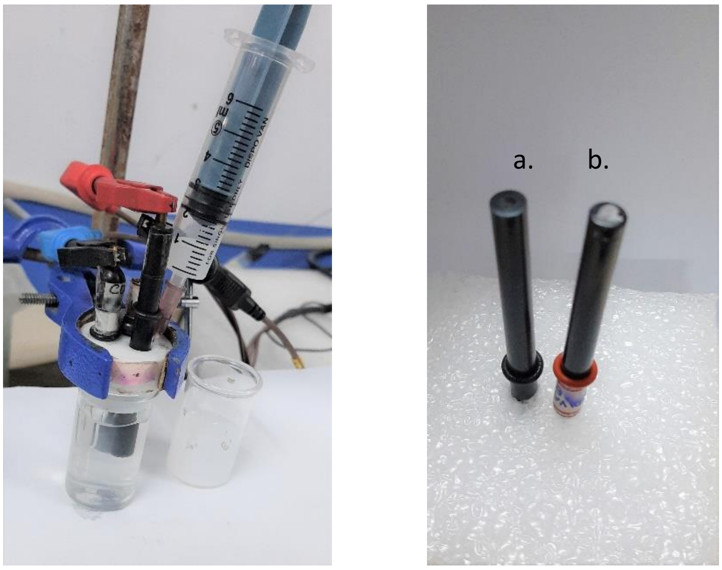

Figure S1. Photographs of (A) a conventional three-electrode system used in this work and (B) GCE/MWCNT@Car-HQ before (a) and after exposure (b) with a 100 µM $AgNO_2$ in the pH 7 PBS. White color precipitate obtained with case (b) electrode indicates liberation of Br- in the reaction medium due to an electrochemical reaction.

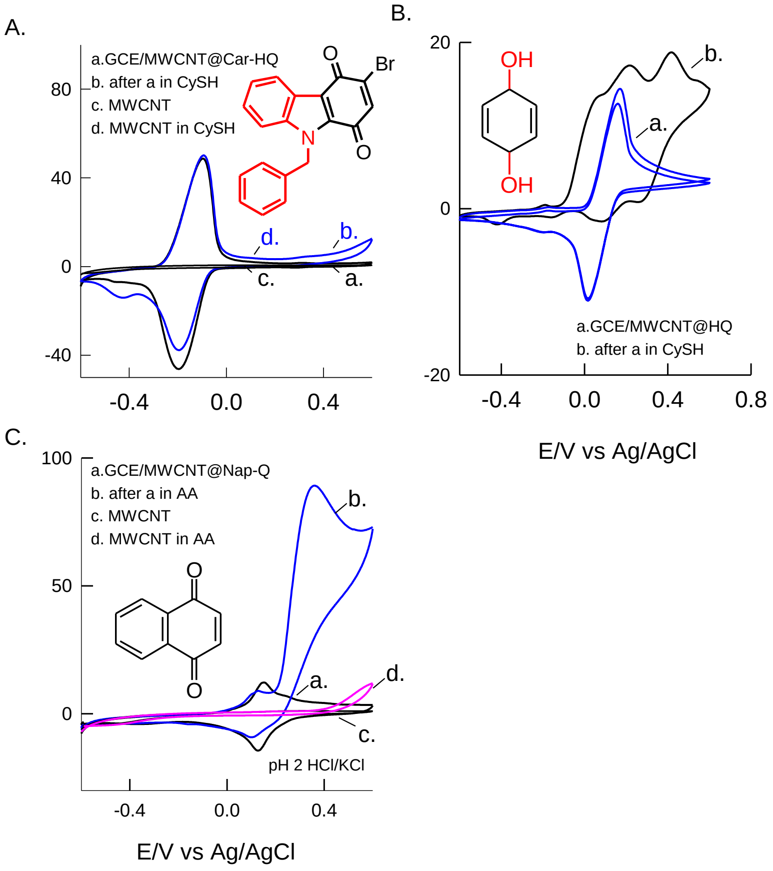

Figure S2. CV responses of various organic compounds immobilized MWCNT modified glassy carbon electrodes without and with added ascorbic acid (AA) and cysteine (CySH) in pH 7 PBS at v=10 mV s^{-1}.

A.

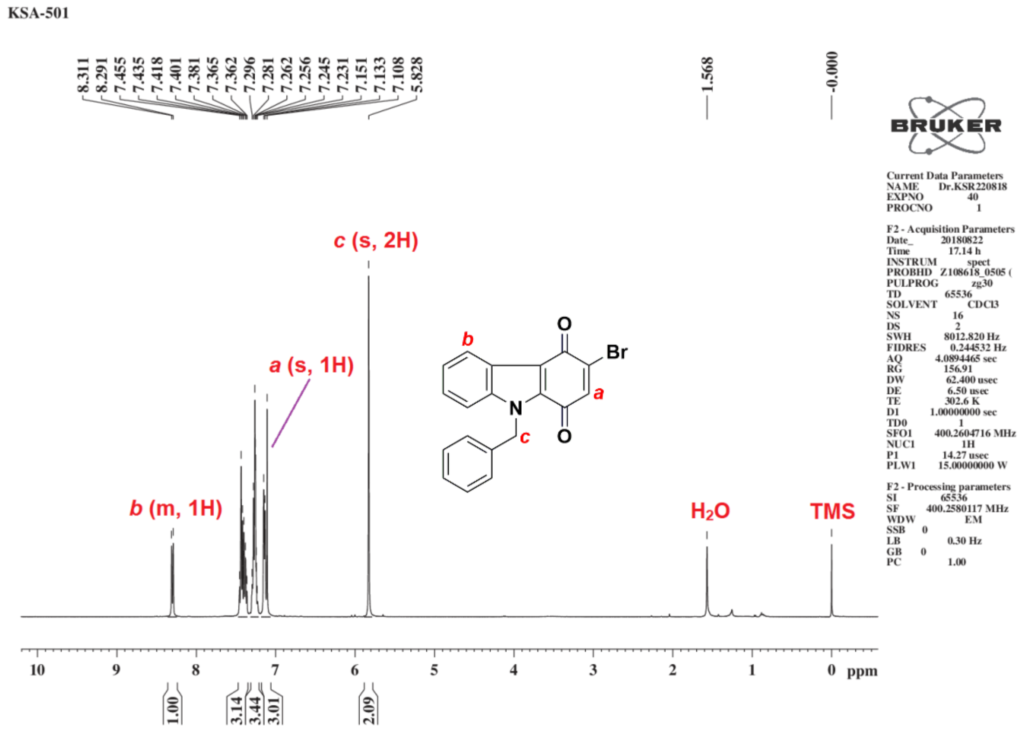
KSA-501
8.311
8.291
7.455
7.435
7.418
7.401
7.381
7.365
7.362
7.296
7.281
7.262
7.256
7.245
7.231
7.151
7.133
7.108
5.828
1.568
-0.000
BRUKER
Current Data Parameters
NAME Dr.KSR220818
EXPNO 40
PROCNO 1
F2 - Acquisition Parameters
Date_ 20180822
Time 17.14 h
INSTRUM spect
PROBHD Z108618_0505 (
PULPROG zg30
TD 65536
SOLVENT CDCl3
NS 16
DS 2
SWH 8012.820 Hz
FIDRES 0.244532 Hz
AQ 4.0894465 sec
RG 156.91
DW 62.400 usec
DE 6.50 usec
TE 302.6 K
D1 1.00000000 sec
TD0 1
SFO1 400.2604716 MHz
NUC1 1H
P1 14.27 usec
PLW1 15.00000000 W
F2 - Processing parameters
SI 65536
SF 400.2580117 MHz
WDW EM
SSB 0
LB 0.30 Hz
GB 0
PC 1.00
c (s, 2H)
a (s, 1H)
b (m, 1H)
H2O
TMS
b
O
Br
a
N
c
O
10
9
8
7
6
5
4
3
2
1
0
ppm
1.00
3.14
3.44
3.01
2.09

B.

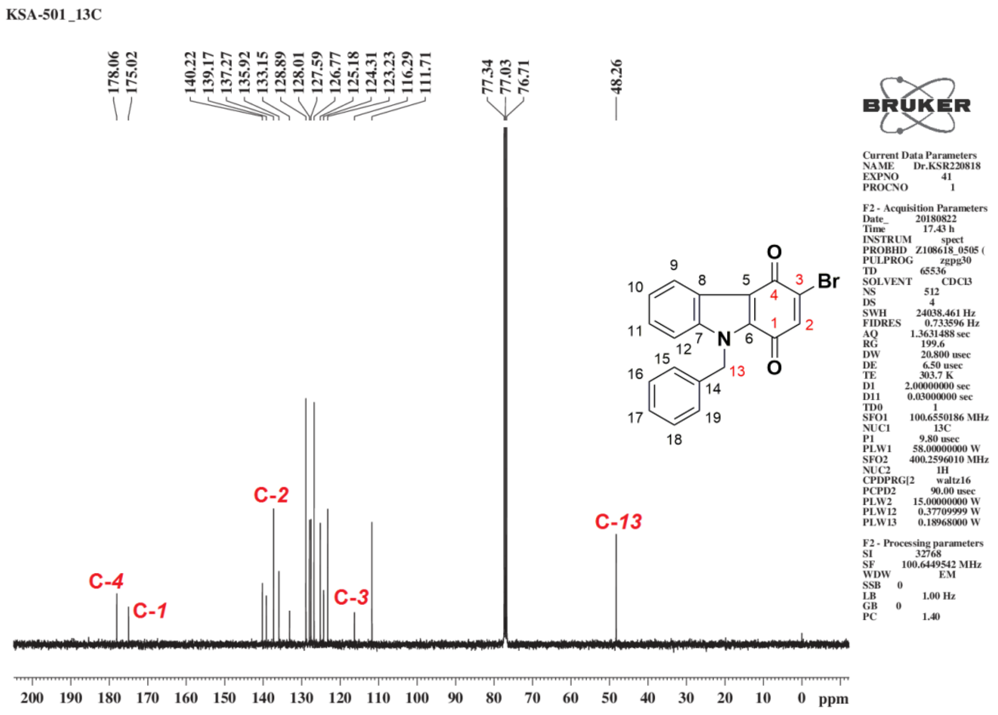
KSA-501_13C
178.06
175.02
140.22
139.17
137.27
135.92
133.15
128.89
128.01
127.59
126.77
125.18
124.31
123.23
116.29
111.71
77.34
77.03
76.71
48.26
BRUKER
Current Data Parameters
NAME Dr.KSR220818
EXPNO 41
PROCNO 1
F2 - Acquisition Parameters
Date_ 20180822
Time 17.43 h
INSTRUM spect
PROBHD Z108618_0505 (
PULPROG zgpg30
TD 65536
SOLVENT CDCl3
NS 512
DS 4
SWH 24038.461 Hz
FIDRES 0.733596 Hz
AQ 1.3631488 sec
RG 199.6
DW 20.800 usec
DE 6.50 usec
TE 303.7 K
D1 2.00000000 sec
D11 0.03000000 sec
TD0 1
SFO1 100.6550186 MHz
NUC1 13C
P1 9.80 usec
PLW1 58.00000000 W
SFO2 400.2596010 MHz
NUC2 1H
CPDPRG[2 waltz16
PCPD2 90.00 usec
PLW2 15.00000000 W
PLW12 0.37709999 W
PLW13 0.18968000 W
F2 - Processing parameters
SI 32768
SF 100.6449542 MHz
WDW EM
SSB 0
LB 1.00 Hz
GB 0
PC 1.40
C-4
C-1
C-2
C-3
C-13
O
Br
N
O
200 190 180 170 160 150 140 130 120 110 100 90 80 70 60 50 40 30 20 10 0 ppm

C.

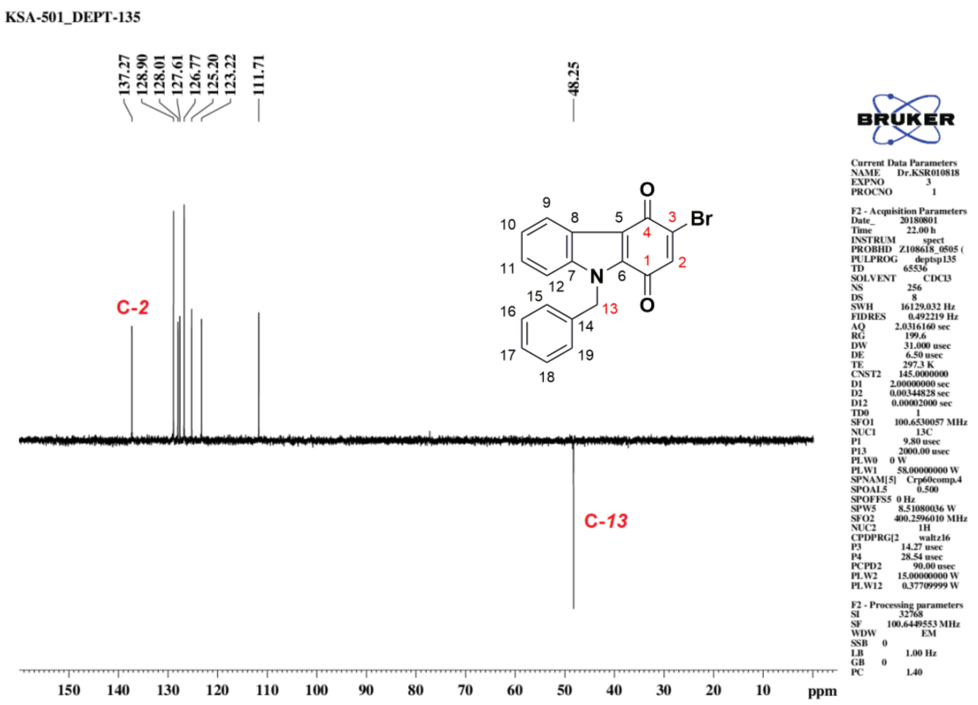

D.

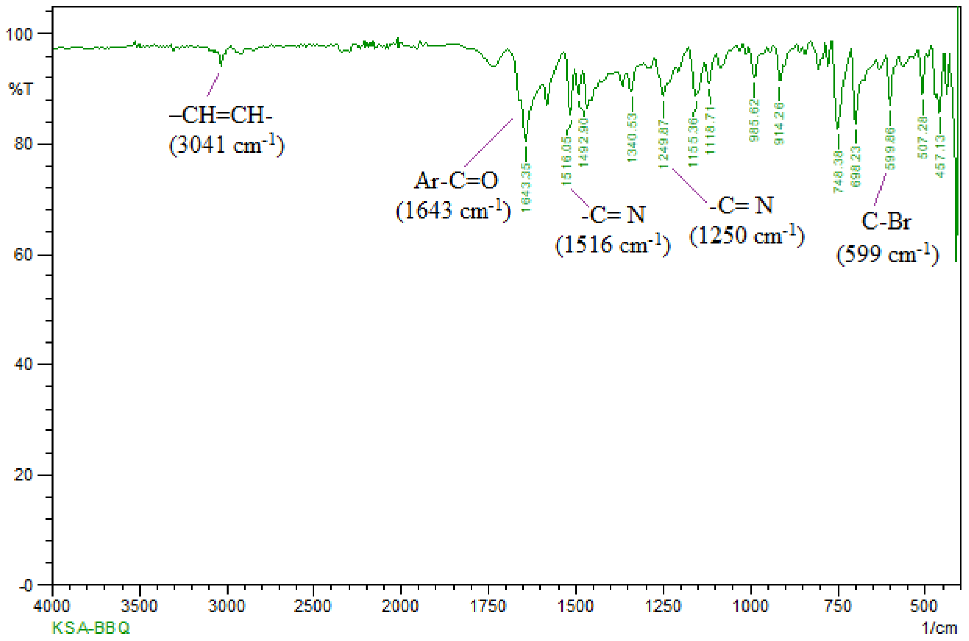

Figure S3. (A-B) are the^{1}H,^{13}C ,DEPT NMR and (D) FT-IR spectra of the compound 9-benzyl-3-bromo-iH-carbazole-1,4(9H)-dione

References

41. Czeskis, B. A. & Wheeler, W. J. Synthesis of β3 adrenergic receptor agonist LY377604 and its metabolite 4-hydroxycarbazole, labeled with carbon-14 and deuterium. *J. Label. Compd. Radiopharm.* **48**, 407–419 (2005).

42. Sissouma, D., Maingot, L., Collet, S. & Guingant, A. Concise and Efficient Synthesis of Calothrixin B. *J. Org. Chem.* **71**, 8384–8389 (2006).

-ooOoo-
